# Genomic Diversity and Resistance Backgrounds of Enterobacteriaceae from Gulls and Coastal Environments

**DOI:** 10.3390/genes17060666

**Published:** 2026-06-07

**Authors:** Vanessa Silva, Micaela Quintelas, Manuela Caniça, Rani Rivière, Patricia Poeta, Gilberto Igrejas

**Affiliations:** 1 CECAV—Veterinary and Animal Research Centre, University of Trás-os-Montes and Alto Douro (UTAD), 5000-801 Vila Real, Portugal; 2 Associate Laboratory for Animal and Veterinary Science (AL4AnimalS), 5000-801 Vila Real, Portugal; micaelaquintelas@gmail.com (M.Q.); manuela.canica@insa.min-saude.pt (M.C.); 3 Microbiology and Antibiotic Resistance Team (MicroART), Department of Veterinary Sciences, University of Trás-os-Montes and Alto Douro (UTAD), 5000-801 Vila Real, Portugal; 4 National Reference Laboratory of Antibiotic Resistances and Healthcare Associated Infections, Department of Infectious Diseases, National Institute of Health Dr. Ricardo Jorge, 1649-016 Lisbon, Portugal; raniriviere1404@gmail.com; 5 Centre for the Studies of Animal Science, Institute of Agrarian and Agri-Food Sciences and Technologies, University of Porto, 4099-002 Porto, Portugal; 6 LAQV-REQUIMTE, Department of Chemistry, NOVA School of Science and Technology, Universidade Nova de Lisboa, 1099-085 Caparica, Portugal; 7 Department of Genetics and Biotechnology, University of Trás-os-Montes and Alto Douro (UTAD), 5000-801 Vila Real, Portugal; 8 Functional Genomics and Proteomics Unit, University of Trás-os-Montes and Alto Douro (UTAD), 5000-801 Vila Real, Portugal

**Keywords:** *Enterobacterales*, gulls, coastal environments, antimicrobial resistance, whole-genome sequencing, *Klebsiella*, *Enterobacter*

## Abstract

**Background/Objectives***: Enterobacterales* are widely distributed in animals and environmental matrices and represent important reservoirs of antimicrobial resistance within a One Health framework. Gulls are particularly relevant because of their synanthropic behavior and frequent contact with anthropogenic waste, which may facilitate the acquisition and dissemination of resistant bacteria. Therefore, this study aimed to investigate the diversity, genomic backgrounds, and resistance determinants of *Enterobacterales* recovered from gulls and coastal environments in Portugal. **Methods:** A total of 265 samples, including seagull feces, saltwater, stagnant water, and sand, were collected from four coastal locations. *Enterobacterales* isolates were recovered and characterized by whole-genome sequencing, and comparative genomic analyses were performed for selected chromosomal beta-lactamase regions. **Results:** *Enterobacterales* were recovered from 41 samples and comprised 11 *Klebsiella* spp., 11 *Leclercia* spp., 9 *Enterobacter* spp., and 3 *Citrobacter* spp. isolates. Isolates were recovered mainly from seagull feces, followed by sand, and Berlenga Grande and Miramar showed the highest diversity. *Klebsiella* spp. and *Enterobacter* spp. concentrated most clinically relevant resistance determinants, including chromosomal *bla*_SHV_-like, *bla*_OXY_-like, and *bla*_ACT_-like genes. By contrast, *Leclercia pneumoniae* was mainly associated with sand and showed a limited resistome, whereas *Citrobacter braakii* carried relevant genes such as *bla*_CMY_ and *qnrB10*. Comparative genomic analysis showed conserved chromosomal contexts surrounding *bla*_SHV_-like, *bla*_OXY_-like, and *bla*_ACT_-like regions. **Conclusions:** These findings indicate that gull-associated fecal contamination may play an important role in the introduction and dissemination of resistant *Enterobacterales* in coastal ecosystems, with subsequent spread to sand and water. Coastal gulls and beach-associated matrices may therefore act as relevant reservoirs and indicators of antimicrobial resistance circulation at the human–environment interface.

## 1. Introduction

Enterobacteriaceae represent a highly diverse bacterial family, exhibiting broad ecological versatility, a wide host range, and significant pathogenic potential for humans, animals, insects, and plants [1]. This family currently includes dozens of genera and hundreds of named species, and members are globally distributed [1]. They are commonly found in soil, water, fruits, meats, eggs, vegetables, grains, flowering plants, and a variety of animals ranging from insects to humans. Due to their rapid generation time, ability to grow on defined media, and ease of genetic manipulation, Enterobacteriaceae have become important models for laboratory studies [1].

Antibiotic resistance, particularly multidrug resistance, has become an increasing global concern among Enterobacteriaceae. These bacteria have developed resistance to many classes of antibiotics, including β-lactams, which remain among the most widely used agents for treating bacterial infections [2]. Continuous exposure to various β-lactams has driven the evolution and dissemination of β-lactamase enzymes, including extended-spectrum β-lactamases (ESBLs), which can hydrolyze third-generation cephalosporins and monobactams but remain inhibited by clavulanic acid [3]. Infections caused by ESBL-producing bacteria are associated with increased mortality, prolonged hospital stays, and higher healthcare costs [2]. The prevalence and phenotypic characteristics of ESBL-producing isolates vary significantly depending on geographical regions, reflecting differences in antibiotic usage and infection control practices [2].

A substantial proportion of resistance in Enterobacteriaceae arises from mobile genetic elements, such as plasmids, which facilitate horizontal gene transfer between different species and even genera [4]. This highlights the environment, including wildlife, as a potential reservoir and source of clinically relevant antimicrobial resistance (AMR) [5]. It is commonly assumed that AMR in wildlife originates from contact with anthropogenic sources, including agricultural runoff and human waste, which introduce resistant bacteria and antibiotics into natural ecosystems [5]. Nevertheless, assigning precise sources and transmission pathways of AMR remains challenging, even in wildlife populations living in close proximity to human activities [6]. Interestingly, resistant bacteria have also been detected in wildlife from remote areas with minimal or no direct exposure to anthropogenic contamination.

The circulation of antimicrobial resistance genes (ARGs) among humans, animals, and the environment exemplifies the interconnected nature of the One Health framework, which emphasizes collaborative efforts across disciplines and sectors to achieve optimal health outcomes for people, animals, and ecosystems [7,8]. Within this framework, the environment represents the most dynamic and complex component, as illustrated by the examples of antibiotic resistance and climate change [9].

Among wild birds, seagulls are particularly noteworthy as potential sentinels for AMR monitoring. These synanthropic birds inhabit not only coastal regions but also inland areas, frequently nesting on buildings, warehouses, and shipyards. They travel long distances in search of food, often visiting landfills, sewage outlets, agricultural lands, and farms, which increases their exposure to human and animal waste [10,11]. By frequenting such environments, gulls can act as vectors, transferring bacteria from contaminated sites to recreational beaches and other public areas [10,11].

Several European studies have reported antimicrobial-resistant Enterobacteriaceae and *Enterobacterales* in wild birds, particularly gulls [11,12,13,14,15,16,17]. Large-scale surveys across Europe showed marked geographical variation in resistant *Escherichia coli* from gulls, with higher resistance levels reported in southern/Iberian regions than in northern Europe [12]. In addition, ESBL-producing *E. coli*, especially CTX-M-type producers, have been detected in gulls from France, Sweden, Portugal, Germany, and Spain, supporting the role of gulls as sentinels of environmental AMR contamination and as potential disseminators of clinically relevant resistance determinants [14,15,16,18,19]. These studies highlight the wild birds’ role as possible bioindicators of environmental AMR pressure. These birds can act both as receivers of antibiotic-resistant bacteria and ARGs (thus serving as sentinels of environmental contamination) and as reservoirs capable of disseminating AMR to different regions and species, including humans and domestic animals. Notably, some gull species have impressive migratory capacities; for example, the Franklin’s gull (*Leucophaeus pipixcan*) migrates from Canada to Chile, potentially spreading AMR across large geographic areas [20,21]. Their presence in urban and rural habitats, combined with widespread dispersal of feces, underscores their relevance in AMR and genetic surveillance and One Health studies [20,21]. Therefore, we aimed to understand the dynamics of Enterobacteriaceae in seagulls and their environments by investigating the antimicrobial resistance and genetic lineages of Enterobacteriaceae isolated from seagulls and beach environments.

## 2. Materials and Methods

### 2.1. Sampling and Bacterial Isolates

Between October 2021 and October 2022, a total of 265 samples were obtained from several locations. These included Berlenga Grande Island (100 samples), Aver-o-Mar beach in Póvoa de Varzim, Porto (55 samples), and the beaches of Matosinhos and Miramar, both located in the Porto region (50 and 60 samples, respectively). Among these samples, 152 were seagull feces, 50 were saltwater, 18 were stagnant water, and 45 were beach sand. Additional information regarding the sampling sites and number of samples is presented in Table 1.

Samples of seawater, sand, stagnant water, and seagull feces were collected using sterile spatulas and transferred into sterile Eppendorf tubes. Fecal samples were identified in the field with the support of an experienced biologist familiar with the local avifauna and with the typical appearance of gull feces. Identification was based on visual characteristics, freshness of the material, and collection from areas where gulls were actively present. As individual defecation events were not systematically observed and the host origin was not confirmed by molecular methods, these samples were considered presumptive gull fecal samples.

For each sampling point, approximately 100 mL of water and 10 g of surface sand were collected. Sand samples were taken from the upper surface layer using sterile spatulas and transferred into sterile containers. Environmental samples were preferentially collected close to visible fecal deposits, within approximately 1 to 2 m, to assess the possible transfer of gull-associated Enterobacteriaceae from fecal material to nearby beach matrices. This targeted approach was intended to evaluate local contamination associated with fecal deposition, rather than the overall background contamination of each beach. Accordingly, the lack of apparently clean control sites, such as sand or water collected away from visible fecal deposits, represents a limitation of the study and should be considered when interpreting the environmental distribution of isolates.

All samples were transported to the laboratory and processed on the same day of collection. Fecal and sand samples were inserted into Brain Heart Infusion (BHI, Liofilchem, Roseto degli Abruzzi, Italy) broth tubes and incubated at 37 °C for 24 h. Water samples were filtered using cellulose nitrate membrane filters with a pore size of 0.45 μm (Whatman, Brentford, UK). The membranes were subsequently placed into tubes containing 5 mL of BHI broth and incubated at 37 °C for 24 h. Following incubation, the enrichment cultures were streaked onto HiChrome *Klebsiella* Selective Agar Base with *Klebsiella* Selective Supplement (HiMedia Laboratories, Mumbai, India) and *Chromogenic Coliform* Agar (OXOID, Bury, UK). All plates were incubated at 37 °C for 24 h. Colonies with morphological characteristics of Enterobacteriaceae were recovered from each plate and further identified by MALDI-TOF (Bruker Daltonics, Bremen, Germany).

### 2.2. Antimicrobial Susceptibility Testing

All isolates were characterized for AMR using the Kirby–Bauer disk diffusion method against 14 antimicrobial agents and according to the EUCAST guidelines [22]. The antibiotics tested were the following: amoxicillin–clavulanic acid (30 µg), ampicillin (10 µg), aztreonam (30 µg), cefotaxime (30 µg), cefoxitin (30 µg), ceftazidime (30 µg), ertapenem (10 µg), streptomycin (10 µg), gentamicin (10 µg), imipenem (10 µg), meropenem (10 µg), tetracycline (30 µg), and trimethoprim–sulfamethoxazole (25 µg).

### 2.3. Whole-Genome Sequencing

Whole-genome sequencing (WGS) was performed for all isolates included in this study. Prior to DNA extraction, the bacterial isolates were cultured on MacConkey agar (Liofilchem) to allow colony differentiation and subsequently subcultured on nutrient agar to obtain pure cultures. Genomic DNA was extracted using an automated magnetic-bead-based method with the MagNA Pure 96 system and the MagNA Pure 96 DNA and Viral NA Small Volume Kit (Roche Diagnostics, Mannheim, Germany), according to the manufacturer’s instructions. DNA concentration was measured using a Qubit™ 4 fluorometer (Thermo Scientific, Waltham, MA, USA). Sequencing libraries were constructed using the Nextera XT library preparation kit (Illumina, San Diego, CA, USA). Whole-genome sequencing was carried out on an Illumina MiSeq platform, producing paired-end reads of 150 bp. The resulting raw sequencing data were processed through a bioinformatics workflow that included read quality assessment, trimming, genome assembly, and downstream genomic analyses.

Quality evaluation and trimming of sequencing reads were conducted using FastQC (v0.11.5) and Trimmomatic (v0.38) [23]. Genome completeness was assessed with BUSCO (v5.5.0_cv1) [24]. De novo genome assembly, species identification, and sequence type determination were performed using INNUca (v4.2.2-02) (https://github.com/B-UMMI/INNUca; accessed 2 July 2025). Species assignment was additionally confirmed through average nucleotide identity (ANI) analysis using FastANI (v1.33), comparing assembled genomes with reference sequences obtained from the National Center for Biotechnology Information (NCBI) GenBank database.

Identification of ARGs was carried out using abriTAMR (v1.0.14) and ABRicate (v1.0.1) (http://github.com/tseemann/abricate; accessed on 2 July 2025). For the ABRicate analysis, multiple public databases were queried, including ARG-ANNOT, ResFinder, CARD, NCBI, PlasmidFinder, and VFDB. Finally, multilocus sequence typing (MLST) was applied to determine the sequence types of the isolates.

Comparative genomic visualization of chromosomal beta-lactamase regions was performed for selected *Enterobacter* spp. and *Klebsiella* spp. isolates. Genome assemblies were annotated with Prokka (v1.15.6), and the coordinates of the target beta-lactamase genes were identified using ABRicate against the NCBI database. For *Enterobacter* spp., regions spanning 10 kb upstream and downstream of *bla*_ACT_-like were extracted from representative isolates. For *Klebsiella* spp., regions spanning 6 kb upstream and downstream of *bla*_SHV_-like or *bla*_OXY_-like were extracted from selected genomes. Comparative visualization was performed using clinker, and figures were exported in SVG format for final editing [25].

## 3. Results and Discussion

### 3.1. Prevalence

A total of 41 Enterobacteriaceae were isolated from the 265 samples collected, corresponding to an overall prevalence of 15.5% (95% CI: 11.6–20.3). Among these, 11 were *Klebsiella* spp. (4.1%; 95% CI: 2.3–7.3), 11 were *Leclercia* spp. (4.1%; 95% CI: 2.3–7.3), nine were *Enterobacter* spp. (3.4%; 95% CI: 1.8–6.3), and three were *Citrobacter* spp. (1.1%; 95% CI: 0.4–3.3). Isolates were recovered from all sample types, with higher absolute numbers observed in seagull feces and sand than in saltwater or stagnant water; however, this distribution was not statistically significant. Berlenga Grande concentrated the highest number and diversity of isolates, followed by Miramar, while Matosinhos and Aver-o-Mar yielded fewer isolates. *Klebsiella* spp. and *Enterobacter* spp. were detected mainly in fecal samples, whereas *L. pneumoniae* was more frequently recovered from sand. *Citrobacter* spp. was less frequent, but it was also detected in both feces and sand. These findings indicate that gull-associated fecal contamination may play an important role in the introduction and dissemination of *Enterobacterales* in the beach environment, with subsequent spread to surrounding matrices such as sand and water. The higher number and diversity of isolates recovered from Berlenga Grande should be interpreted with caution, since this site also had the largest number of samples collected. Nevertheless, local ecological conditions may have contributed to the observed distribution. Differences among sampling sites may reflect variation in gull abundance and activity, intensity of fecal deposition, beach morphology, environmental persistence in sand and water, and the degree of anthropogenic influence, including recreational pressure, urban runoff, wastewater-related contamination, or proximity to human activities. In more isolated areas, such as Berlenga Grande, gull density and repeated fecal deposition may be particularly relevant drivers of bacterial introduction into the surrounding environment, whereas in more urbanized beaches, local anthropogenic inputs may also contribute to the maintenance or selection of resistant Enterobacterales. However, because specific socio-economic, land-use, and physicochemical environmental variables were not measured, these explanations remain hypothetical and should be further investigated in future studies. Other studies have reported the presence of Enterobacteriaceae in gull feces, including *Klebsiella* spp. [18,26,27,28,29], *Enterobacter* spp. [29,30], *Citrobacter* spp. [28,29,30], and *Leclercia* spp. [31] from different countries and continents. However, direct comparison of prevalence among countries is limited by differences in sampling strategies, sample types, bacterial selection methods, and identification approaches. Compared with previous European studies, our isolates showed a less extensive acquired resistome, since major ESBL genes such as *bla*_CTX-M_ and carbapenemase genes were not detected. This contrasts with reports from gulls in Portugal, France, Sweden, Germany, and Spain, where ESBL or carbapenemase-producing Enterobacteriaceae have been described [11,12,13,14,15,16,18,19,29]. Nevertheless, the detection of chromosomal beta-lactamase backgrounds, quinolone-associated genes, plasmid replicons, and metal resistance genes in the present study supports the relevance of gulls and coastal matrices as indicators of AMR circulation at the human–environment interface.

### 3.2. Klebsiella spp.

A total of 11 *Klebsiella* spp. isolates were characterized by WGS. Nine isolates were identified as *K. pneumoniae*, whereas VS3383 and VS3382 were assigned to *Klebsiella michiganensis* and *Klebsiella oxytoca*, respectively. Most isolates were recovered from seagull feces, with only VS3381 being isolated from sand. The predominance of *K. pneumoniae* suggests that this species was the main representative of the genus in the sampled coastal environment, while the detection of *K. michiganensis* and *K. oxytoca* indicates additional genomic diversity within this group.

The *K. pneumoniae* isolates showed a relatively conserved resistome, mainly characterized by the presence of *bla*_SHV_, *oqxA*/*oqxB*, and *fosA* (Table 2). Among them, *bla*_SHV-108_ was the most frequent beta-lactamase and was detected in six isolates. Two isolates (VS3380 and VS3381) carried *bla*_SHV-1_, whereas isolate VS3375 harbored *bla*_SHV-11_. Genes associated with fosfomycin resistance were detected in all *K. pneumoniae* isolates, corresponding to *fosA* in all isolates except *fosA7* in isolate VS3381. In addition, all nine *K. pneumoniae* isolates carried *oqxA* and *oqxB*, while *acrD* was only detected in three isolates. The recurrent detection of *oqxA* and *oqxB* in our isolates may explain, at least in part, the common background of reduced susceptibility to quinolones and other compounds. The OqxAB system is a multidrug efflux pump that has been associated with decreased susceptibility to quinolones/fluoroquinolones and chloramphenicol, among other agents. Accordingly, the widespread distribution of *oqxAB* in our *Klebsiella* spp. isolates suggests that efflux-mediated mechanisms may contribute to the resistance phenotype observed in this genus, even if they do not act as the sole determinant of resistance [32,33,34]. This shows that the *K. pneumoniae* isolates shared a common resistance background, mostly involving beta-lactams, fosfomycin, and efflux-mediated decreased susceptibility to quinolones and other compounds. The resistome detected in our *K. pneumoniae* isolates differed from that previously described in gull-associated isolates from other regions. In one study conducted in gulls, *K. pneumoniae* isolates showed a resistome mainly characterized by the presence of *bla*_TEM-1_, *bla*_CTX-M-3_, *bla*_SHV-187_, and *bla*_CTX-M-15_, whereas *fosA* was detected in all isolates [26]. By contrast, *K. pneumoniae* isolates identified in the present study showed a more conserved resistance background with no *bla*_CTX-M_ genes being detected. These differences suggest that gull-associated *K. pneumoniae* populations may vary considerably according to geographical location and local selective pressures. Our isolates also appeared to show a less extensive resistance profile than those described in a previous study performed in gulls from the Lisbon area, Portugal, in 2020 [29]. In that study, only one *Klebsiella* spp. isolate showed resistance to fosfomycin, which contrasts with our results. This finding may indicate differences in the circulation of fosfomycin resistance determinants among gull-associated *Klebsiella* spp. populations from distinct Portuguese coastal regions.

PointFinder analysis revealed recurrent chromosomal mutations in genes related to efflux regulation and outer membrane permeability, particularly *acrR*, *ompK36*, and *ompK37.* Six *K. pneumoniae* isolates (VS3373, VS3374, VS3376, VS3377, VS3378, and VS3379) shared the same mutational profile, including substitutions in *acrR* (P161R, G164A, F172S, R173G, L195V, F197I, and K201M), *ompK36* (N49S, L59V, L191S, F207W, A217S, N218H, D224E, L228V, E232R, and T254S), and *ompK37* (I70M and I128M). A second profile was observed in VS3375, VS3380, and VS3381, which shared the same substitutions in *acrR* but fewer changes in *ompK36* and *ompK37*. These mutations are more likely to act as accessory mechanisms rather than as primary resistance determinants, but their recurrence suggests a conserved chromosomal background among most *K. pneumoniae* isolates [35,36,37].

Despite the relative homogeneity of the resistome, *K. pneumoniae* isolates were not clonal. Four isolates belonged to ST505 and shared K64, *wzi*64, and O1αβ,2α, indicating a close genomic relatedness. VS3378 and VS3379 both belonged to ST889 and were associated with K54, *wzi*117, and O1αβ,2α. The remaining *K. pneumoniae* isolates belonged to ST1426, ST13, and ST252. Therefore, although the same core resistance determinants were repeatedly identified, the typing data showed the circulation of different *K. pneumoniae* lineages in the sampled sites. Furthermore, ST505 was only detected in Berlenga Grande, whereas ST889 was identified in both Matosinhos and Miramar, suggesting differences in the local distribution of *Klebsiella* spp. lineages among the sampled beaches. Interestingly, ST505, which was the most common lineage detected in our study, appears to be rare, since only a few studies have reported *K. pneumoniae* ST505, namely from human infections in Mexico, Chile, and China [38,39,40]. To the best of our knowledge, this lineage has not yet been reported in animals. ST889, which was detected in two isolates, also appears to be a rare lineage. To date, it has been described in the dairy production process and has also been associated with hypervirulence [41]. ST1426 has been previously reported in wastewater, human infections, and pigs [42,43,44]. In addition, ST13 seems to be relatively common in Portuguese hospitals, where it is usually associated with carbapenem resistance, which contrasts with our findings [45,46,47].

Virulence-associated determinants were scarce in the *K. pneumoniae* group. Most isolates had a virulence score of 0 and showed no evidence of hypermucoidy. The only exception was VS3380, which belonged to ST13 and had a Kleborate virulence score of 2. This score was associated with the detection of the yersiniabactin locus, assigned as yersiniabactin sequence type 289 (YbST289), and the colibactin locus, assigned as colibactin sequence type 79 single-locus variant (CbST79-1LV). The *ybt* locus encodes the siderophore yersiniabactin, involved in iron acquisition, whereas the *clb*/*pks* locus encodes the genotoxin colibactin [48]. Thus, although most *K. pneumoniae* isolates appeared to carry mainly resistance-associated traits, the presence of VS3380 suggests that coastal environments may also harbor isolates with additional virulence-related potential.

Differences were also observed regarding plasmid content and metal resistance genes. The ST505 isolates were generally associated with *fieF* and small Col-type plasmids, while VS3374, VS3376, and VS3377 additionally carried IncFII(K) and IncFIB(pQil) replicons. The two ST889 isolates carried several genes linked to copper and silver resistance, namely the *pco* and *sil* operons, together with IncFIB(K). VS3381 showed the broadest adaptive profile, carrying multiple *pco*, *sil*, *ncr*, and *ars* genes, as well as IncFIB(K), IncFII(K), and repB(R1701) replicons. These findings suggest that, besides ARGs, some isolates also carried traits that may contribute to persistence in polluted or metal-impacted environments, since heavy metals can co-select for antimicrobial resistance and favor the maintenance of resistant bacteria in environmental reservoirs. This is particularly relevant in coastal ecosystems, which are often exposed to anthropogenic inputs, including metal contamination [49,50,51,52].

The two non-*pneumoniae* isolates clearly differed from the remaining genomes. VS3383, identified as *K. michiganensis*, carried *bla*_OXY-1-1_, *fosA*, *aph(3′)-Ia*, and *acrD*. It was also the only isolate carrying an acquired aminoglycoside resistance gene. In addition, it harbored IncFIB(K) (pCAV1099-114) and a large number of copper, silver, and arsenic resistance genes. VS3382, identified as *K. oxytoca*, carried *bla*_OXY-2-4_, *oqxA*, *oqxB*, and *fosA* and belonged to ST199. Compared with the other isolates, it showed a more divergent chromosomal profile, particularly in *ompK36*, and a much more limited set of metal resistance genes. The presence of *bla*_OXY_ genes in these two isolates is in agreement with their species assignment and clearly separates them from the *K. pneumoniae* group [53,54].

**Figure 1:**
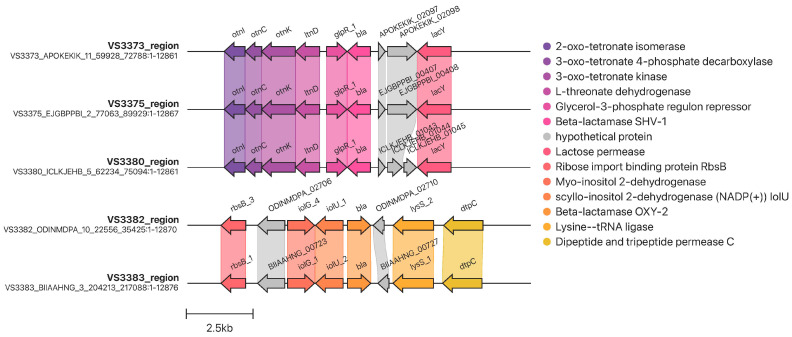
Comparative genomic analysis of the chromosomal *bla*_SHV_-like and *bla_O_*_XY_-like regions in selected *Klebsiella* spp. isolates. The *K. pneumoniae* genomes shared a *bla*_SHV_-like chromosomal background, whereas *K. oxytoca* and *K. michiganensis* carried distinct *bla*_OXY_-like regions.

Comparative genomic analysis of the chromosomal *bla*_SHV_-like and *bla*_OXY_-like regions revealed a clear separation between the *K. pneumoniae* isolates and the two non-pneumoniae genomes (Figure 1). *K. pneumoniae* isolates shared a *bla*_SHV_-like chromosomal background with a broadly conserved surrounding genomic organization, despite variation in the specific SHV-like alleles identified. By contrast, *K. oxytoca* and *K. michiganensis* carried distinct *bla*_OXY_-like regions, highlighting a different chromosomal beta-lactamase background from that observed in the *K. pneumoniae* group. This interpretation agrees with previous studies indicating that the SHV family originated from the chromosomal beta-lactamase background of *K. pneumoniae*, while *bla*_OXY_ represents the intrinsic chromosomal beta-lactamase of the *K. oxytoca* complex, including *K. michiganensis* [55,56].

### 3.3. Leclercia spp.

A total of 11 *Leclercia* spp. isolates were identified in this study, and all were assigned to *L. pneumoniae*. Most isolates were recovered from sand (*n* = 7), followed by seagull feces (*n* = 4) and one stagnant water sample. Regarding their geographical distribution, most isolates were obtained in Berlenga Grande (*n* = 9), whereas two isolates were recovered in Aver-o-Mar and one in Miramar. Therefore, *L. pneumoniae* was mainly associated with Berlenga Grande and was more frequently detected in sand than in fecal samples. Although *L. pneumoniae* showed a limited resistome when compared with the other *Enterobacterales* identified in this study, its detection remains relevant due to the relatively high number of isolates recovered. In fact, *Leclercia* spp. was one of the most frequently detected genera, which suggests that this species may be well adapted to the sampled coastal environment. The predominance of *L. pneumoniae* in sand samples, particularly in Berlenga Grande, further supports the idea that this species may persist in the surrounding beach environment rather than being restricted to fecal deposition (Table 3). Therefore, even though it did not represent a major reservoir of clinically relevant resistance determinants in our collection, its frequent occurrence makes it an interesting genus from an ecological point of view.

The resistome of the *L. pneumoniae* isolates was limited. Genes associated with fosfomycin resistance were identified in seven isolates, corresponding to *fosA* and *fosA8*. The remaining isolates did not carry other ARGs. *L. pneumoniae* is likely intrinsically resistant to fosfomycin, as members of the genus are known to carry chromosomal *fosA*-like genes. Therefore, the detection of *fosA/fosA8*-related genes in these isolates probably reflects the intrinsic genomic background of this genus rather than the acquisition of a major resistance determinant [57,58].

The metal resistance gene *fieF* was detected in almost all isolates and was therefore the most conserved genomic trait in this group. In addition, isolate VS3394, recovered from seagull feces in Miramar, also carried *arsB*, making it the only *L. pneumoniae* isolate with an additional metal resistance-associated determinant. VS3394 was also the only isolate carrying a plasmid, namely IncFII(p14)_1_p14. Therefore, although the *L. pneumoniae* showed very limited antimicrobial resistance, some degree of genomic variability was observed regarding metal resistance genes and plasmid content.

### 3.4. Enterobacter spp.

A total of nine *Enterobacter* spp. isolates were characterized by WGS. These isolates belonged to six different species, namely *Enterobacter hormaechei* (*n* = 4), *Enterobacter kobei* (*n* = 2), and single isolates of *Enterobacter bugandensis*, *Enterobacter mori*, and *Enterobacter ludwigii* (Table 4). Most isolates were recovered from seagull feces (*n* = 6), whereas two were isolated from saltwater and one from sand. Regarding their geographical distribution, four isolates were obtained in Berlenga Grande, two in Matosinhos, two in Miramar, and one in Aver-o-Mar. Therefore, Berlenga Grande was the location with the highest number of *Enterobacter* isolates, while the genus was also detected in all sample types analyzed, including feces, saltwater, and sand.

**Figure 2:**
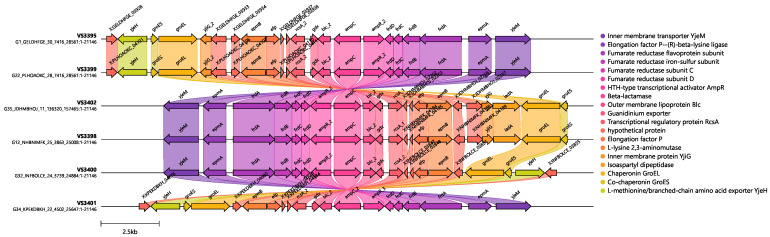
Comparative genomic analysis of the chromosomal *bla*_ACT_-like region in selected *Enterobacter* spp. isolates. The genomic context surrounding *bla*_ACT_-like was highly conserved across isolates, supporting its location within a typical chromosomal *Enterobacter* spp. background.

Overall, the *Enterobacter* spp. isolates showed a relatively conserved resistome, mainly characterized by the presence of *bla*_ACT_-type beta-lactamases together with *oqxA*, *oqxB*, and *fosA*/*fosA2*/*fosA7*. With the exception of VS3397, all isolates carried an ACT-family AmpC beta-lactamase. Different ACT variants were identified, namely *bla*_ACT-14_, *bla*_ACT-47_, *bla*_ACT-23_, *bla*_ACT-77_, *bla*_ACT-22_, and *bla*_ACT-28_. The predominance of *bla*_ACT_-like genes among our *Enterobacter* spp. isolates is in agreement with previous studies showing that members of the *Enterobacter cloacae* complex typically carry an inducible chromosomal AmpC β-lactamase, which represents an important intrinsic mechanism of resistance to β-lactams. In this genus, ACT-type enzymes are among the most frequently reported AmpC variants, and different ACT alleles may be identified within closely related *Enterobacter* lineages. Therefore, the detection of several *bla*_ACT_ variants in our isolates is consistent with the known chromosomal β-lactamase background of *Enterobacter* spp. and supports the idea that AmpC production is a major feature of resistance in this group [59,60,61]. Comparative genomic analysis of the *bla*_ACT_-like region showed a largely conserved chromosomal context among the selected *Enterobacter* spp. isolates, despite variation in the ACT-like alleles identified (Figure 2). In particular, the genomic organization surrounding *bla*_ACT_-like remained highly similar across isolates, with conservation of the flanking genes and overall synteny. This finding supports the idea that these genes are inserted within a typical chromosomal *Enterobacter* background and suggests that the main variation occurs at the allele level rather than through major structural differences in the surrounding locus. Therefore, the comparative genomic analysis reinforces the interpretation that the *bla*_ACT_-like genes detected in our isolates are consistent with the intrinsic AmpC background commonly described in *Enterobacter* spp. [62,63]. *oqxA* and *oqxB* were detected in all isolates except VS3397, and a fosfomycin-associated gene was present in all isolates except VS3397. The presence of *fosA/fosA2*-like genes in most of our *Enterobacter* spp. isolates is also supported by previous studies showing that FosA-family enzymes are important determinants of fosfomycin resistance in Enterobacterales. In particular, *fosA2* was originally described in an environmental isolate of *Enterobacter cloacae*, where it was shown to confer fosfomycin resistance, and later studies confirmed that FosA-family genes are widely distributed among clinically relevant Gram-negative bacteria [64,65]. Among the isolates analyzed, VS3400 was the most notable in terms of acquired resistance genes, since it was the only one carrying *qnrE3* in addition to *bla*_ACT-77_, *oqxA*, *oqxB*, and *fosA*. The presence of *qnrE3* suggests an additional quinolone resistance mechanism beyond the efflux-associated profile shared by most isolates, since Qnr proteins are known to protect DNA gyrase and topoisomerase IV from quinolone inhibition, and *qnrE3* has been shown to confer decreased susceptibility to fluoroquinolones [66,67]. By contrast, VS3397, identified as *E. kobei*, did not carry any of the ARGs, making it the only isolate without an evident acquired resistance genotype.

No virulence gene, hypermucoidy-associated genotype, or virulence score was recorded for any of the isolates. Therefore, in this collection, the genomic characterization of *Enterobacter* spp. was mainly marked by resistance and adaptation-associated traits rather than by recognized virulence-associated determinants.

All isolates except VS3397 carried *fieF*, indicating that this metal-associated gene was almost ubiquitous among the *Enterobacter* genomes. Additional metal resistance genes were, however, unevenly distributed. VS3397 and VS3401 carried several genes related to copper and silver resistance, including the *pco* and *sil* operons. VS3402 showed the broadest adaptive profile, harboring the *ter*, *ars*, *sil*, and *pco* genes in addition to *fieF*. These findings suggest that some *Enterobacter* spp. isolates may possess an enhanced capacity to persist in environments under metal-associated selective pressure. Since metal resistance genes may co-occur with antimicrobial resistance determinants and contribute to co-selection, these adaptive traits may also favor the maintenance of resistant lineages in coastal ecosystems exposed to anthropogenic inputs [68,69,70].

Plasmid content also varied among isolates. Three genomes carried IncFIB-related plasmids, all of which corresponded to fecal isolates from *E. hormaechei*. VS3401 carried IncFII(pECLA) and IncFIA(HI1), while VS3402 showed the highest plasmid diversity, including IncFIB(K), IncFII(pECLA), Col(pHAD28), IncR, Col440II, and ColRNAI. By contrast, no plasmids were recorded for the remaining isolates. The higher plasmid content observed in VS3402 and some *E. hormaechei* isolates may favor the maintenance and dissemination of both resistance and adaptation-associated genes.

The species distribution among locations was also noteworthy. Berlenga Grande had the highest number of *Enterobacter* spp. isolates and included three species, namely *E. hormaechei*, *E. bugandensis*, and one additional *E. hormaechei* lineage. Matosinhos included *E. mori* and *E. ludwigii*, while Miramar included *E. kobei* and *E. hormaechei*. Aver-o-Mar yielded only one isolate identified as *E. kobei*. This distribution suggests that different beaches may harbor different *Enterobacter* species and lineages, although the number of isolates is still limited. Most isolates were recovered from seagull feces, which supports the idea that gulls may contribute to the dissemination of *Enterobacter* spp. in these coastal settings. The detection of *Enterobacter* spp. in sand and saltwater also suggests that fecal contamination may favor the spread of these bacteria to the surrounding beach environment.

### 3.5. Citrobacter spp.

A total of three *Citrobacter* spp. isolates were identified in this study. One isolate was identified as *Citrobacter gillenii* and two as *C. braakii* (Table 5). The isolates were recovered from different sample types and locations, namely seagull feces from Aver-o-Mar (*C. gillenii*), sand from Berlenga Grande (*C. braakii*), and seagull feces from Matosinhos (*C. braakii*). Therefore, although the number of isolates was low, *Citrobacter* was detected in both feces and the surrounding beach environment and in three different sampling locations. The genomic analysis showed distinct resistance profiles between the *Citrobacter* species. The *C. gillenii* isolate carried only *bla*_GIL-1_, together with several genes associated with metal resistance, namely *fieF* and the *ars* operon. By contrast, the two *C. braakii* isolates carried more relevant resistance determinants. Isolate VS3405, recovered from sand, harbored *bla*_CMY-100_ and *qnrB10*, in addition to *fieF*, whereas VS3406, recovered from seagull feces, carried *bla*_CMY_ and several arsenic-associated genes together with *fieF*. Thus, the two *C. braakii* isolates showed a more clinically relevant resistome than the *C. gillenii* isolate, mainly due to the presence of *bla*_CMY_ and, in one case, *qnrB10*. The detection of *bla*_CMY_ in both *C. braakii* isolates is noteworthy, since this gene is associated with AmpC-mediated resistance to beta-lactams and may contribute to resistance to extended-spectrum cephalosporins [71,72]. In addition, the presence of *qnrB10* in VS3405 suggests an additional mechanism of quinolone resistance [66]. By contrast, the *C. gillenii* isolate carried *bla*_GIL-1_, a chromosome-encoded narrow-spectrum class A β-lactamase that is likely related to the intrinsic β-lactamase background of this species and appears to be of lower clinical relevance than *bla*_CMY_ [73].

## 4. Conclusions

This study highlights the role of gulls and coastal environments as relevant interfaces for the circulation of *Enterobacterales* with antimicrobial resistance potential. Although the isolates belonged to different genera and showed distinct ecological and genomic patterns, the findings suggest that gull-associated fecal contamination may contribute to the introduction and dissemination of resistant bacteria in beach ecosystems. *Klebsiella* spp. and *Enterobacter* spp. were the most relevant genera from a clinical perspective, whereas *L. pneumoniae* appeared to be more associated with environmental persistence, and *C. raakii*, although less frequent, carried noteworthy resistance determinants. Overall, these results support the inclusion of wildlife-associated coastal environments in One Health AMR surveillance strategies.

## Figures and Tables

**Table 1:** Number of samples collected by sample type and location.

Location	Seagull Feces	Saltwater	Stagnant Water	Sand	Total
Berlenga Grande	62	20	3	15	100
Aver-o-Mar	30	10	5	10	55
Miramar	30	10	10	10	60
Matosinhos	30	10	-	10	50
Total	152	50	18	45	265

**Table 2:** Genomic features of *Klebsiella* spp. isolates from gulls and coastal environments, including resistance, virulence, molecular typing, plasmid content, and metal resistance genes.

Isolate	Origin	Location	Species	Antimicrobial Resistance Genes	Virulence Score	MLST	Metal Resistance	Plasmids
VS3373	Seagull feces	Berlenga Grande	*K. pneumoniae*	*bla*_SHV-108_, *oqxB19*, *oqxA*, *fosA*	0	ST505	*fieF*	Col(pHAD28), Col440I
VS3374	Seagull feces	Berlenga Grande	*K. pneumoniae*	*bla*_SHV-108_, *oqxB19*, *oqxA*, *fosA*	0	ST505	*fieF*	Col(pHAD28), Col440I, IncFII(K), IncFIB(pQil)
VS3375	Seagull feces	Berlenga Grande	*K. pneumoniae*	*bla*_SHV-11_, *oqxB14*, *oqxA*, *fosA*	0	ST1426	*fieF*	
VS3376	Seagull feces	Berlenga Grande	*K. pneumoniae*	*bla*_SHV-108_, *oqxB19*, *oqxA*, *fosA*	0	ST505	*fieF*	Col(pHAD28), Col440I, IncFII(K), IncFIB(pQil)
VS3377	Seagull feces	Berlenga Grande	*K. pneumoniae*	*bla*_SHV-108_, *oqxB19*, *oqxA*, *fosA*	0	ST505	*fieF*	Col(pHAD28), Col440I, IncFII(K), IncFIB(pQil)
VS3378	Seagull feces	Matosinhos	*K. pneumoniae*	*bla*_SHV-108_, *oqxB*, *oqxA*, *fosA*	0	ST889	*pcoS*, *pcoR*, *pcoD*, *pcoC*, *pcoB*, *pcoA*, *silP*, *silA*, *silB*, *silF*, *silC*, *silR*, *silS*, *silE*	IncFIB(K)
VS3379	Seagull feces	Miramar	*K. pneumoniae*	*bla*_SHV-108_, *oqxB*, *oqxA*, *fosA*	0	ST889	*fieF*, *silE*, *silS*, *silR*, *silC*, *silF*, *silB*, *silA*, *silP*, *pcoA*, *pcoB*, *pcoC*, *pcoD*, *pcoR*, *pcoS*	IncFIB(K)
VS3380	Seagull feces	Miramar	*K. pneumoniae*	*bla*_SHV-1_, *oqxB25*, *oqxA*, *fosA*	2 (YbST 289, CbST 79-1LV)	ST13	*fieF*	ColRNAI, Col440II
VS3381	Sand	Miramar	*K. pneumoniae*	*bla*_SHV-1_, *oqxB19*, *oqxA*, *fosA7*	-	ST252	*acrD*, *pcoS*, *pcoR*, *pcoD*, *pcoC*, *pcoB*, *pcoA*, *silP*, *silA*, *silB*, *silF*, *silC*, *silR*, *silS*, *silE*, *ncrA*, *ncrB*, *ncrC*, *ncrY*, *arsR*, *arsD*, *arsA*, *arsB*, *arsC*	IncFIB(K), IncFII(K), repB(R1701), IncFII(K)
VS3382	Seagull feces	Miramar	*K. oxytoca*	*bla*_OXY-2-4_, *oqxB*, *oqxA*, *fosA*	-	ST199	*arsB*, *arsC*, *fieF*	-
VS3383	Seagull feces	Berlenga Grande	*K. michiganensis*	*bla*_OXY-1-1_, *fosA*, *aph*(3’)-Ia	*ybtP*, *ybtQ*	ST11	*fieF*, *arsR*, *arsD*, *arsA*, *arsB*, *arsC*, *pcoS*, *pcoR*, *pcoD*, *pcoC*, *pcoB*, *pcoA*, *silP*, *silA*, *silB*, *silF*, *silC*, *silR*, *silS*, *silE*	IncFIB(K) (pCAV1099-114)

**Table 3:** Genomic features of *Leclercia* spp. isolates from gulls and coastal environments.

Isolate	Origin	Location	Species	Antimicrobial Resistance Genes	Metal Resistance
VS3384	Sand	Berlenga Grande	*Leclercia pneumoniae*	*fosA*	*fieF*
VS3385	Seagull feces	Aver-o-Mar	*Leclercia pneumoniae*	*fosA8*	*fieF*
VS3386	Seagull feces	Aver-o-Mar	*Leclercia pneumoniae*	*fosA8*	*fieF*
VS3387	Stagnant water	Berlenga Grande	*Leclercia pneumoniae*	*-*	*fieF*
VS3388	Sand	Berlenga Grande	*Leclercia pneumoniae*	*fosA8*	*fieF*
VS3389	Sand	Berlenga Grande	*Leclercia pneumoniae*	*fosA*	*fieF*
VS3390	Seagull feces	Berlenga Grande	*Leclercia pneumoniae*	*-*	*fieF*
VS3391	Sand	Berlenga Grande	*Leclercia pneumoniae*	*fosA8*	*-*
VS3392	Sand	Berlenga Grande	*Leclercia pneumoniae*	*-*	*fieF*
VS3393	Sand	Berlenga Grande	*Leclercia pneumoniae*	*-*	*fieF*
VS3394	Seagull feces	Miramar	*Leclercia pneumoniae*		*fieF*, *arsB*

**Table 4:** Genomic features of *Enterobater* spp. isolates from gulls and coastal environments.

Isolate	Origin	Location	Species	Antimicrobial Resistance Genes	Metal Resistance	Plasmids
VS3395	Saltwater	Berlenga Grande	*E. hormaechei*	*bla*_ACT-14_, *oqxA*, *oqxB*, *fosA2*	*fieF*	
VS3396	Seagull feces	Berlenga Grande	*E. hormaechei*	*bla*_ACT-47_, *oqxA*, *oqxB*, *fosA2*	*fieF*	IncFIB(pHCM2)
VS3397	Seagull feces	Aver-o-Mar	*E. kobei*	-	*fie*F, *pcoE*, *pcoS*, *pcoR*, *pcoD*, *pcoC*, *pcoB*, *pcoA*, *silP*, *silA*, *silB*, *silF*, *silC*, *silR*, *silS*, *silE*	
VS3398	Seagull feces	Berlenga Grande	*E. bugandensis*	*bla*_ACT-47_, *oqxA*, *oqxB*, *fosA2*	*fieF*	
VS3399	Seagull feces	Berlenga Grande	*E. hormaechei*	*bla*_ACT-23_, *oqxA*, *oqxB*, *fosA*	*fieF*	IncFIB(pHCM2)_1_pHCM2
VS3400	Sand	Matosinhos	*E. mori*	*bla*_ACT-77_, *oqx*A, *oqx*B, *qnr*E3, *fos*A	*fieF*	
VS3401	Saltwater	Matosinhos	*E. ludwigii*	*bla*_ACT-22_, *oqxA*, *oqxB*, *fosA*	*fieF*, *silR*, *silA*, *silE*, *silS*, *silR*, *silC*, *silF*, *silB*, *silA*, *silP*, *pcoA*, *pcoC*, *pcoD*, *pcoR*, *pcoS*	IncFII(pECLA)_1_pECLA, IncFIA(HI1)_1_HI1
VS3402	Seagull feces	Miramar	*E. kobei*	*bla*_ACT-28_, *oqxA*, *oqxB*, *fosA7*	*fieF*, *terD*, *terZ*, *terW*, *arsR*, *arsD*, *arsA*, *arsB*, *arsC*, *arsR*, *arsD*, *arsA*, *arsB*, *arsC*, *silE*, *silS*, *silR*, *silC*, *silF*, *silB silA*, *silP*, *pcoA*, *pcoB*, *pcoC*, *pcoD*, *pcoR pcoS*	IncFIB(K), IncFII(pECLA), Col(pHAD28), IncR_1, Col440II_1, ColRNAI_1
VS3403	Seagull feces	Miramar	*E. hormaechei*	*bla*_ACT-47_, *oqxA*, *oqxB*, *fosA*	*fieF*	IncFIB(pB171)_1_pB171

**Table 5:** Genomic features of *Citrobacter* spp. isolates from gulls and coastal environments.

Isolate	Origin	Location	Species	Antimicrobial Resistance Genes	Metal Resistance
VS3404	Seagull feces	Aver-o-Mar	*C. gillenii*	*bla*_GIL-1_	*fieF*, *arsR*, *arsD*, *arsA*, *arsB*, *arsC*
VS3405	Sand	Berlenga Grande	*C. braakii*	*bla*_CMY-100_, *qnrB10*	*fieF*
VS3406	Seagull feces	*Matosinhos*	*C. braakii*	*bla*_CMY_	*fieF*, *arsC*, *arsB*, *arsA*, *arsD*, *arsR*

## Data Availability

The original contributions presented in this study are included in the article. Further inquiries can be directed to the corresponding authors.
